# Whole-genome and pangenome insights into *Mycobacterium colombiense* clinical isolates from human infections

**DOI:** 10.7717/peerj.20716

**Published:** 2026-02-02

**Authors:** Chutinthorn Oungbamnet, Yothin Hinwan, Nut Nithimongkolchai, Vorthon Sawaswong, Suwalak Chitcharoen, Kiatichai Faksri, Auttawit Sirichoat

**Affiliations:** 1 Department of Microbiology, Faculty of Medicine, Khon Kaen University, Khon Kaen, Thailand; 2 Research and Diagnostic Center for Emerging Infectious Diseases (RCEID), Khon Kaen University, Khon Kaen, Thailand; 3 Department of Biochemistry, Faculty of Science, Mahidol University, Bangkok, Thailand

**Keywords:** *Mycobacterium colombiense*, Pangenome analysis, Genetic diversity, Antimicrobial resistance, Virulence factors

## Abstract

**Background:**

Nontuberculous mycobacteria are opportunistic pathogens which cause infections in various tissues, with the *Mycobacterium avium* complex (MAC) being a major cause of pulmonary diseases. Among MAC members, *Mycobacterium colombiense* is a clinically significant species with recognized pathogenic potential; however, studies on its genomic structure and genetic diversity remain limited.

**Methods:**

This study investigated the drug susceptibility profiles and performed whole-genome sequencing of 12 clinical *M. colombiense* isolates from the Clinical Microbiology Laboratory at Srinagarind Hospital, Khon Kaen University, Khon Kaen, Thailand.

**Results:**

Based on minimum inhibitory concentration values, moxifloxacin resistance was most prevalent (100%), followed by linezolid (90%), clarithromycin (30%), and amikacin (30%). The presence of antibiotic resistance genes and virulence factors, including ESX secretion systems and efflux pumps, highlights the bacterium’s potential for immune evasion and survival under stress. Single nucleotide polymorphism-based phylogenetic analysis revealed a close genetic relatedness between the isolates. Pangenome analysis of 16 *M. colombiense* genomes (12 newly sequenced and four publicly available) identified 7,771 gene clusters, comprising 4,468 core genes, 1,834 accessory genes, and 1,469 unique genes, supporting a closed pangenome structure and indicating evolutionary conservation and low genetic variability among isolates.

**Conclusions:**

This study provides valuable insight into the genomic diversity, antimicrobial resistance profiles, and virulence potential of *M. colombiense* isolates. These findings enhance understanding of the pathogen and may inform clinical management, targeted diagnostic development, and therapeutic strategies.

## Introduction

Nontuberculous mycobacteria (NTM) are mycobacterial species other than those responsible for tuberculosis and leprosy, excluding members of the *Mycobacterium tuberculosis* complex, *Mycobacterium leprae*, and *Mycobacterium lepromatosis*. NTM are opportunistic pathogens widely distributed in natural environments (including water, soil, animals, and plants) capable of infecting various tissues and organs, with pulmonary involvement the most common clinical manifestation (Gu et al., 2023). NTM infections can present as chronic pulmonary disease, lymphadenitis, or cutaneous and disseminated infections, particularly in immunocompromised individuals. Among NTMs, the *Mycobacterium avium* complex (MAC) is most frequently associated with pulmonary disease. The MAC comprises 12 closely related species differing in host specificity, disease severity, virulence factors, and immune responses (Gonzalez-Perez et al., 2016). Notable members include *M. avium*, *M. intracellulare*, and the focus of this study, *M. colombiense*.

*M. colombiense* is a recently described member of the MAC. It is a non-motile, acid-fast, rod-shaped bacterium which typically forms rough, non-pigmented colonies within three weeks of incubation. The organism was first isolated from the blood of four HIV-coinfected patients in Colombia in 1995 (Bouam et al., 2017) and its formal classification was established in 2006. Since its discovery, reports of *M. colombiense* infections have been relatively rare. Due to the limited use of molecular diagnostics, the species was often misidentified as other MAC members, leading to an underrepresentation of its prevalence (Vuorenmaa et al., 2009). Recent molecular studies have revealed that, while its prevalence remains low, *M. colombiense* constitutes a clinically significant proportion of MAC isolates in certain geographic regions (Monteiro et al., 2024; Qin & Tang, 2024). *M. colombiense* has been implicated in disseminated infections involving the lungs, lymph nodes, bones, and joints, often with systemic symptoms such as fever and lymphadenopathy (Tang et al., 2023). Species identification relies primarily on molecular techniques, particularly 16S rRNA gene sequencing which reveals a distinct sequence signature and a novel 16S–23S rRNA internal transcribed spacer sequence designated as the MAC-X sequevar (Gonzalez-Perez et al., 2011; Murcia et al., 2006).

Although advances in molecular diagnostics have improved *M. colombiense* identification, distinguishing MAC species remains challenging. For example, Sanger sequencing of 16S rRNA in previous studies has yielded ambiguous results such as double peaks, suggesting mixed infections or the presence of multiple pathogenic species (Church et al., 2020). Recent genome analyses have provided further insight into the pathogenic mechanisms of *M. colombiense*, revealing deletions in genes involved in the biosynthesis of virulence-associated lipids such as p-HBA/PDIM/PGL, PLC, SL-1, and HspX, as well as the absence of the ESX-1 secretion system (Gonzalez-Perez et al., 2016). Nevertheless, comprehensive studies on *M. colombiense* genetic diversity and genome architecture remain scarce.

Whole-genome sequencing, combined with bioinformatics analysis, plays a pivotal role in elucidating the genetic basis of bacterial virulence, antimicrobial resistance, and evolutionary dynamics (Tang et al., 2023). Among comparative genomic tools, pangenome analysis has emerged as a powerful approach for investigating genetic diversity across multiple strains of a bacterial species. This method classifies genes into three categories: core genes (present in all genomes), accessory genes (shared among some but not all genomes), and unique genes (strain-specific). The composition of the pangenome—whether open or closed—provides insight into its genomic plasticity and evolutionary dynamics (Kim et al., 2020; Lim et al., 2021). A closed pangenome indicates genetic stability; an open pangenome reflects ongoing gene acquisition and diversification.

Despite the utility of pangenome analysis, its application to *M. colombiense* remains largely unexplored. This study therefore investigated the drug susceptibility profiles and genetic diversity of *M. colombiense* clinical isolates from human infections and characterized their genome structure through comprehensive pangenome analysis, with the aim of contributing to a better understanding of the evolutionary biology, epidemiology, and potential therapeutic targets of the pathogen.

## Materials and Methods

### Bacterial isolates

*M. colombiense* clinical isolates were obtained from the collection at the Clinical Microbiology Laboratory at Srinagarind Hospital, Khon Kaen University, Khon Kaen, Thailand. These isolates were collected from patients diagnosed with MAC infections caused by NTM. Diagnosis was made in accordance with the guidelines of the American Thoracic Society and the Infectious Diseases Society of America (Griffith et al., 2007). All MAC isolates were initially identified using the INNO-LiPA MYCOBACTERIA v2 line-probe assay (Fujirebio, Ghent, Belgium) and subsequently sub-cultured on Löwenstein–Jensen medium for further analysis.

The study protocol was approved by the Institutional Review Board of the Khon Kaen University Ethics Committee in Human Research (no. HE671519; approval date: September 24, 2024). All experiments were conducted in accordance with the Declaration of Helsinki and ICH Good Clinical Practice guidelines. Informed consent was waived for the use of medical data as patient information was anonymized and de-identified prior to analysis.

### Drug susceptibility testing

Minimum inhibitory concentrations (MICs) were determined using the Sensititre™ Myco SLOMYCOI assay (TREK Diagnostic Systems, West Sussex, UK) following the manufacturer’s protocol. The assay included serial two-fold dilutions of 13 lyophilized antimicrobial agents: clarithromycin (0.06–64 μg/mL), amikacin (1–64 μg/mL), rifabutin (0.25–8 μg/mL), rifampicin (0.12–8 μg/mL), ethambutol (0.5–16 μg/mL), trimethoprim/sulfamethoxazole (0.12/2.38–8/152 μg/mL), ciprofloxacin (0.12–16 μg/mL), moxifloxacin (0.12–8 μg/mL), ethionamide (0.3–20 μg/mL), isoniazid (0.25–8 μg/mL), doxycycline (0.12–16 μg/mL), linezolid (1–64 μg/mL), and streptomycin (0.5–64 μg/mL). MIC interpretations followed the Clinical and Laboratory Standards Institute (CLSI) guidelines (CLSI, 2018).

### Genomic DNA extraction and whole-genome sequencing

Genomic DNA was extracted using the cetyl-trimethyl-ammonium bromide-sodium chloride method (Larsen et al., 2007), followed by RNase treatment and ethanol precipitation for purification. DNA concentration and quality were assessed using a NanoDrop spectrophotometer (Thermo Fisher Scientific, Carlsbad, CA, USA). High-quality DNA was sent to NovogeneAIT (Hong Kong) for whole-genome sequencing. Paired-end libraries were prepared and sequenced on the Illumina HiSeq 2500 platform with 150-bp read lengths. Sequence data are deposited in the Sequence Read Archive under BioProject accession no. PRJNA1271787.

### Species identification and genome assembly

Raw reads were quality checked using FastQC version 0.11.9 (Andrews, 2010). Adapter sequences and low-quality reads were removed using Trimmomatic version 0.39 (Bolger, Lohse & Usadel, 2014). High-quality paired-end reads were *de novo* assembled using Unicycler version 0.4.8 (Wick et al., 2017). Assemblies were scaffolded using RagTag version 2.1.0 (Alonge et al., 2022) and polished with Pilon version 1.24 (Walker et al., 2014) to improve base accuracy. Assembly quality was evaluated using QUAST version 5.3.0 (Gurevich et al., 2013). Genome completeness and contamination were evaluated using CheckM version 1.2.3 (Parks et al., 2015), applying thresholds of >97% completeness and <3% contamination. Final species identification and genome validation were performed using NTM-Profiler version 0.2.0 (Phelan, 2021), the Type Strains Genome Sever (TYGS) (Meier-Kolthoff et al., 2022), Pathogenwatch (https://pathogen.watch/), and GTDB-Tk (Chaumeil et al., 2022). Pairwise average nucleotide identity (ANI) was calculated using FastANI (Jain et al., 2018) for accurate taxonomic classification. ANI-based clustering and heatmap visualization were generated using ANIclustermap version 1.4.0 (Shimoyama, 2022).

### Genome characterization

Gene prediction and functional annotation were performed using the RAST toolkit (RASTtk) through the Bacterial and Viral Bioinformatics Resource Center (BV-BRC) with a comprehensive genome analysis pipeline (Olson et al., 2023). Antibiotic resistance genes (ARGs) and virulence factors were identified using ABRicate version 0.7 (Seemann, 2020) with default parameters (a minimum sequence identity threshold of 80% and a coverage of 80%). The Comprehensive Antibiotic Resistance Database (CARD) was used to detect resistance genes and the Virulence Factor Database (VFDB) was used to identify virulence-associated genes. ARGs were identified using Resistance Gene Identifier (RGI) version 6.0.5 restricted to “Perfect and Strict hits only” for high-confidence predictions against the most recent version of the CARD (Alcock et al., 2023). The RGI predicts resistomes by utilizing curated homology and single nucleotide polymorphism (SNP) models to detect both acquired resistance genes and chromosomal resistance mechanisms. CRISPR loci and CRISPR-associated (Cas) proteins were detected using CRISPRCasFinder (Couvin et al., 2018) with high-sensitivity parameters to identify both confirmed and putative CRISPR arrays, as well as Cas subtypes.

### Detection of genetic mutations associated with drug resistance

Genetic mutations associated with drug resistance were analyzed in key target genes, including 16S rRNA (*rrs*), 23S rRNA (*rrl*), *gyrA*, *gyrB*, large ribosomal protein L3 (*rplC*), and large ribosomal protein L4 (*rplD*). The *rrs* gene is the resistance target for amikacin, whereas *rrl* is the target for clarithromycin. The *gyrA* and *gyrB* genes serve as targets for moxifloxacin, and *rplC* and *rplD* correspond to linezolid. Gene sequences were extracted from the assembled *M. colombiense* genomes using BLASTn followed by BEDtools getfasta, and subsequently aligned with the reference genome *M. colombiense* CECT 3050 (accession no. NZ_AFVW02000005.1) using the MUSCLE algorithm in MEGA (Kumar et al., 2024) to identify mutations associated with resistance.

### SNP-based phylogenetic analysis

SNP-based phylogenetic analysis was conducted to infer evolutionary relationships among *M. colombiense* isolates. SNP calling was performed using Snippy version 4.6.0 (Seemann, 2015) which aligned reads to the *M. colombiense* reference genome CECT 3035 (NZ_CP020821.1). Core genome SNPs were extracted using Snippy-core, generating a core alignment of 5,581,643 bp, of which 147,025 positions were identified as polymorphic. Recombination was filtered using Gubbins version 3.1.6 (Croucher et al., 2015), resulting a final non-recombinant alignment of 111,537 SNP positions used for downstream phylogenetic reconstruction. All tools were run with default parameters unless otherwise specified. Maximum likelihood trees were constructed using IQ-TREE version 2.0.7 (Minh et al., 2020) with 1,000 bootstrap replicates and the optimal substitution model selected using ModelFinder Plus. The phylogenetic tree was visualized using iTOL version 7 (Letunic & Bork, 2021). Pairwise SNP distance matrices were analyzed using snp-dists version 0.8.2 (Seemann, Klötzl & Page, 2021).

### Public genome retrieval and species confirmation

The 18 publicly available genome assemblies were retrieved from the National Center for Biotechnology Information (https://www.ncbi.nlm.nih.gov/datasets/genome/) (accessed February 2025). Detailed information is provided in Table 1. All genomes were quality checked using CheckM, applying thresholds of >97% completeness and <3% contamination. Species confirmation was performed using NTM-Profiler, TYGS, Pathogenwatch, GTDB-Tk, and FastANI. The publicly available genomes identified as *M. colombiense* were combined with the 12 clinical isolates from this study for pangenome analysis.

**Table 1:** Genome information of the 30 *Mycobacterium colombiense* isolates, including those newly sequenced in the current study and the publicly available genomes retrieved from the National Center for Biotechnology Information.

Isolate	Size (bp)	Read depth^a^	GC content (%)	Accession number	Number of contig	N50/L50^b^	Country
MCO062281265	5,749,731	231×	68.01	SRR33823402	12	5,744,256/1	Thailand
MCO102081859	5,602,039	263×	68.09	SRR33823401	3	5,599,316/1	Thailand
MCO021680268	5,747,785	149×	67.97	SRR33823398	5	5,740,878/1	Thailand
MCO031580717	5,892,330	141×	67.85	SRR33823397	3	5,888,367/1	Thailand
MCO051681085	5,892,050	146×	67.85	SRR33823396	3	5,888,087/1	Thailand
MCO051681160	5,892,002	137×	67.85	SRR33823395	3	5,888,039/1	Thailand
MCO051681265	5,746,932	146×	67.96	SRR33823394	3	5,649,464/1	Thailand
MCO061681546	5,726,541	136×	67.96	SRR33823393	3	5,123,345/1	Thailand
MCO061481970	5,891,326	145×	67.85	SRR33823392	3	5,887,363/1	Thailand
MCO061481978	5,890,968	143×	67.85	SRR33823391	3	5,887,005/1	Thailand
MCO101582585	5,750,826	144×	67.97	SRR33823400	5	5,740,878/1	Thailand
MCO121383869	5,638,640	132×	68.06	SRR33823399	3	5,634,808/1	Thailand
CECT 3035 (reference)	5,578,571	NA	68.09	GCA_000222105.4	4	5,576,643/1	Colombia
TKK-01-0051	6,044,235	NA	67.73	GCA_000661085.1	2	6,042,881/1	South Africa
852002-1834_SCH5396731	5,555,215	NA	67.22	GCA_001665835.1	33	5,494,652/1	South Africa
E2464	5,826,525	NA	67.34	GCA_001667905.1	50	5,745,615/1	Cambodia
E1334	5,561,780	NA	67.55	GCA_001672755.1	37	5,505,303/1	Cambodia
1211504.4	5,844,955	NA	67.43	GCA_001673005.1	116	5,695,033/1	Mozambique
1245759.5	5,668,613	NA	67.48	GCA_001673015.1	76	5,546,326/1	Mozambique
1245829.5	5,758,743	NA	67.42	GCA_001673075.1	48	5,684,243/1	Mozambique
1165916.7	5,705,292	NA	67.52	GCA_001673085.1	77	5,616,652/1	Mozambique
1164983.0	5,729,797	NA	67.42	GCA_001673175.1	53	5,592,831/1	Mozambique
1245197.5	5,643,029	NA	67.52	GCA_001673195.1	53	5,592,831/1	Mozambique
1137323.0	5,967,299	NA	67.21	GCA_001673505.1	42	5,648,100/1	Mozambique
NS-7390	5,615,519	NA	67.51	GCA_001953985.1	20	5,583,716/1	India
IS-1176	5,566,184	NA	67.56	GCA_001954055.1	46	5,522,015/1	India
IS-2214	5,572,614	NA	67.55	GCA_001954075.1	34	5,517,866/1	India
IS-576	5,787,997	NA	67.48	GCA_001954115.1	31	5,553,009/1	India
230	5,669,125	NA	67.94	GCA_023218095.1	10	5,606,113/1	China
CIP108962T	5,571,935	NA	68.08	GCA_965137175.1	93	5,625,163/1	France

**Notes:**

bp, base pairs; NA, not applicable.

a Read depth is calculated as (total number of reads × average read length)/genome size.

b The N50 length, which is defined as the shortest sequence length at 50% of the genome.

b The L50 count, which is defined as the smallest number of contigs whose length sum produces N50.

### Pangenome analysis

All *M. colombiense* genomes were re-annotated using Prokka version 1.14.6 (Seemann, 2014) to generate GFF3 files. Pangenome analysis was conducted using Roary version 3.12.0 (Page et al., 2015) with default parameters, including a 95% identity threshold for BLASTp and a core gene definition of ≥99% presence across genomes. Partial coding sequences were excluded and translated protein sequences were used for downstream analysis. Core genome alignments were refined using MAFFT (Katoh & Standley, 2013) for improved alignment accuracy. The gene presence–absence matrix and summary statistics were generated. Genes were categorized as core (present in all genomes), soft-core (present in most but not all genomes), accessory (present in several genomes), or unique (strain-specific). Visualization of the pangenome analysis was performed using the Python script [roary_plots.py] and the R program with the ggplot2 package. Functional annotation of core, accessory, and unique genes was performed using the Clusters of Orthologous Groups (COGs) system *via* the Pan-Explorer platform (Dereeper, Summo & Meyer, 2022), allowing for comparative analysis of gene functions related to metabolic pathways and functional roles.

### Statistical analysis

Statistical analysis was performed using IBM SPSS Statistics for Windows, version 28.0 (IBM Corp, Armonk, NY, USA). Descriptive statistics were used to summarize the study population characteristics. Normality of the data distribution was evaluated using the Shapiro–Wilk test. Continuous variables with a normal distribution are presented as the mean ± standard deviation, followed by the range (minimum–maximum), while non-normally distributed data are presented as the median. Categorical variables are reported as frequencies and percentages.

## Results

### Characteristics of the studied isolates

A total of 12 *M. colombiense* clinical isolates were identified using bioinformatics tools, including NTM-Profiler, TYGS, Pathogenwatch, GTDB-Tk, and FastANI (Table S1). Most isolates (75%) were obtained from male patients with a mean age of 44.25 ± 15.33 years (range: 27–79 years). Isolates originated from various clinical specimens, including sputum, tracheal suction, skin, pus, synovial fluid, and others (Tables 1, S2).

Drug susceptibility testing was performed on 10 of the 12 isolates using the Sensititre™ Myco SLOMYCOI assay (Table 2), with two isolates excluded due to inadequate growth. MIC breakpoints for amikacin, clarithromycin, linezolid, and moxifloxacin were interpreted according to CLSI guidelines. Results revealed universal resistance to moxifloxacin (100%) and high resistance to linezolid (90%). Moderate resistance was observed for clarithromycin and amikacin (30% each). The MIC50 and MIC90 values demonstrated that resistance to moxifloxacin was consistent at ≥8 µg/mL. Linezolid had elevated MICs at 32 µg/mL (MIC50) and ≥64 µg/mL (MIC90). Susceptibility to clarithromycin was variable (MIC50 of 4 µg/mL and MIC90 of 64 µg/mL); amikacin demonstrated intermediate efficacy (MIC50 of 16 µg/mL and MIC90 of 64 µg/mL).

**Table 2:** Minimum inhibitory concentration (MIC) values for 13 drugs tested against the *Mycobacterium colombiense* clinical isolates.

Isolate	Drug^a^ (MIC as µg/mL)												
	AMI	CIP	CLA	DOX	EMB	ETH	INH	LZD	MXF	RFB	RIF	STR	SXT
MCO021680268	64	≥16	4	≥16	≥16	≥20	≥8	32	4	2	≥8	≥64	4
MCO031580717	8	16	64	16	16	20	8	32	8	1	8	64	8
MCO051681085	64	≥16	≥64	≥16	≥16	≥20	≥8	≥64	≥8	≥8	≥8	64	≥8
MCO051681160	≥64	≥16	≥64	≥16	≥16	≥20	≥8	≥64	≥8	8	≥8	32	≥8
MCO051681265	16	≥16	8	≥16	4	20	≥8	32	≥8	1	≥8	32	8
MCO061681546	32	≥16	1	≥16	8	≥20	≥8	32	≥8	2	≥8	32	8
MCO061481970	16	16	4	16	16	20	8	32	8	4	8	64	8
MCO061481978	16	16	4	16	16	10	8	64	8	8	8	32	8
MCO101582585	32	16	1	≥16	8	≥20	≥8	32	≥8	1	≥8	16	2
MCO121383869	16	16	1	16	16	20	8	16	8	2	8	32	8
**Breakpoint (µg/mL)**	**≥64**	**NA**	**≥32**	**NA**	**NA**	**NA**	**NA**	**≥32**	**≥4**	**NA**	**NA**	**NA**	**NA**
MIC50^b^	16	16	4	≥16	16	20	≥8	32	8	2	≥8	32	8
MIC90^b^	64	≥16	64	≥16	≥16	≥20	≥8	≥64	≥8	8	≥8	64	≥8
Range	8 –≥64	16 –≥16	1 –≥64	16 –≥16	4 –≥16	10 –≥20	8 –≥8	16 –≥64	4 –≥8	1 –≥8	8 –≥8	16 –≥64	2 –≥8
Resistant (%)	3 (30)	NA	3 (30)	NA	NA	NA	NA	9 (90)	10 (100)	NA	NA	NA	NA

**Notes:**

a AMI, amikacin; CIP, ciprofloxacin; CLA, clarithromycin; DOX, doxycycline; EMB, ethambutol; ETH, ethionamide; INH, isoniazid; LZD, linezolid; MXF, moxifloxacin; RFB, rifabutin; RIF, rifampicin; STR, streptomycin; SXT, trimethoprim/sulfamethoxazole.

b MIC50 or MIC90, the lowest concentration of an antimicrobial agent inhibiting the growth of 50% or 90% of the tested bacterial population.

NA, not applicable as the Clinical and Laboratory Standards Institute guidelines have not established a breakpoint for this drug.

### Gene prediction and functional annotation

The average genome size of the 12 isolates was 5.79 Mbp (Table 3). The number of predicted coding sequences ranged from 5,350 to 5,665, with an average of 5,471. All isolates had a GC content of approximately 67.84%. Each genome contained 3 rRNA genes, while tRNA gene numbers ranged from 49 to 51.

**Table 3:** General genome features of the 12 *Mycobacterium* *colombiense* clinical isolates.

Feature/protein coding genes	Isolate											
	MCO062281265	MCO102081859	MCO021680268	MCO031580717	MCO051681085	MCO051681160	MCO051681265	MCO061681546	MCO061481970	MCO061481978	MCO101582585	MCO121383869
Source	NA	Sputum	Fluid	Wound swab	Elbow (pus)-left	Synovial fluid	Sputum	Tissue	Chest (pus)	Inguinal abscess	Sputum	Sputum
Collection date (d/m/y)	8/6/2022	27/10/2020	2/2/2016	3/10/2015	5/5/2016	13/5/2016	25/5/2016	24/6/2016	7/6/2014	9/6/2014	5/10/2015	6/12/2013
Genome size (bp)	5,749,731	5,602,039	5,747,785	5,892,330	5,892,050	5,892,002	5,746,932	5,726,541	5,891,326	5,890,968	5,750,826	5,638,640
GC content (%)	68.01	68.09	67.97	67.85	67.85	67.85	67.96	67.96	67.85	67.85	67.97	68.06
No. of contigs	12	3	5	3	3	3	3	3	3	3	5	3
Contigs N50 (bp)	5,744,256	5,599,316	5,740,878	5,888,367	5,888,087	5,888,039	5,649,464	5,123,345	5,887,363	5,887,005	5,740,878	5,634,808
Contigs L50	1	1	1	1	1	1	1	1	1	1	1	1
No. of CDSs	5,532	5,350	5,478	5,662	5,665	5,665	5,472	5,457	5,658	5,654	5,486	5,374
No. of subsystems	256	259	271	270	270	270	271	271	270	270	271	269
tRNAs	47	47	49	49	49	49	49	49	49	49	49	51
rRNA	3	3	3	3	3	3	3	3	3	3	3	3
**No. of antibiotic resistance genes**												
Victors	4	4	4	4	4	4	4	4	4	4	4	4
CARD	11	13	13	13	13	13	13	13	13	13	13	13
PATRIC	43	45	46	45	45	45	46	46	45	45	48	46
**No. of virulence factors**												
Victors	98	105	107	106	106	106	108	108	106	106	108	106
VFDB	13	19	19	18	19	18	22	19	18	18	19	18
PATRIC	149	164	168	166	166	166	172	169	166	166	172	166
CRISPR loci	7	6	6	8	8	9	6	6	8	8	7	8

**Notes:**

d/m/y, date/month/year; bp, base pairs; CDSs, coding DNA sequences; NA, not applicable.

The N50 length, which is defined as the shortest sequence length at 50% of the genome.

The L50 count, which is defined as the smallest number of contigs whose length sum produces N50.

Victors, CARD, PATRIC, and VFDB refer to the corresponding antibiotic resistance and virulence and pathogenicity factor databases using the RAST toolkit *via* the Bacterial and Viral Bioinformatics Resource Center platform.

Genome annotation using the RASTtk on the BV-BRC platform identified consistent numbers of annotated antibiotic resistance and virulence genes across isolates. The Victors database reported four resistance genes per genome, the CARD database detected 11–13 genes, and the PathoSystems Resource Integration Center (PATRIC) database identified the highest number (43–46 genes). Similarly, virulence gene counts varied by database: Victors reported 98–108, VFDB identified 13–22, and PATRIC detected 149–172.

**Figure 1:**
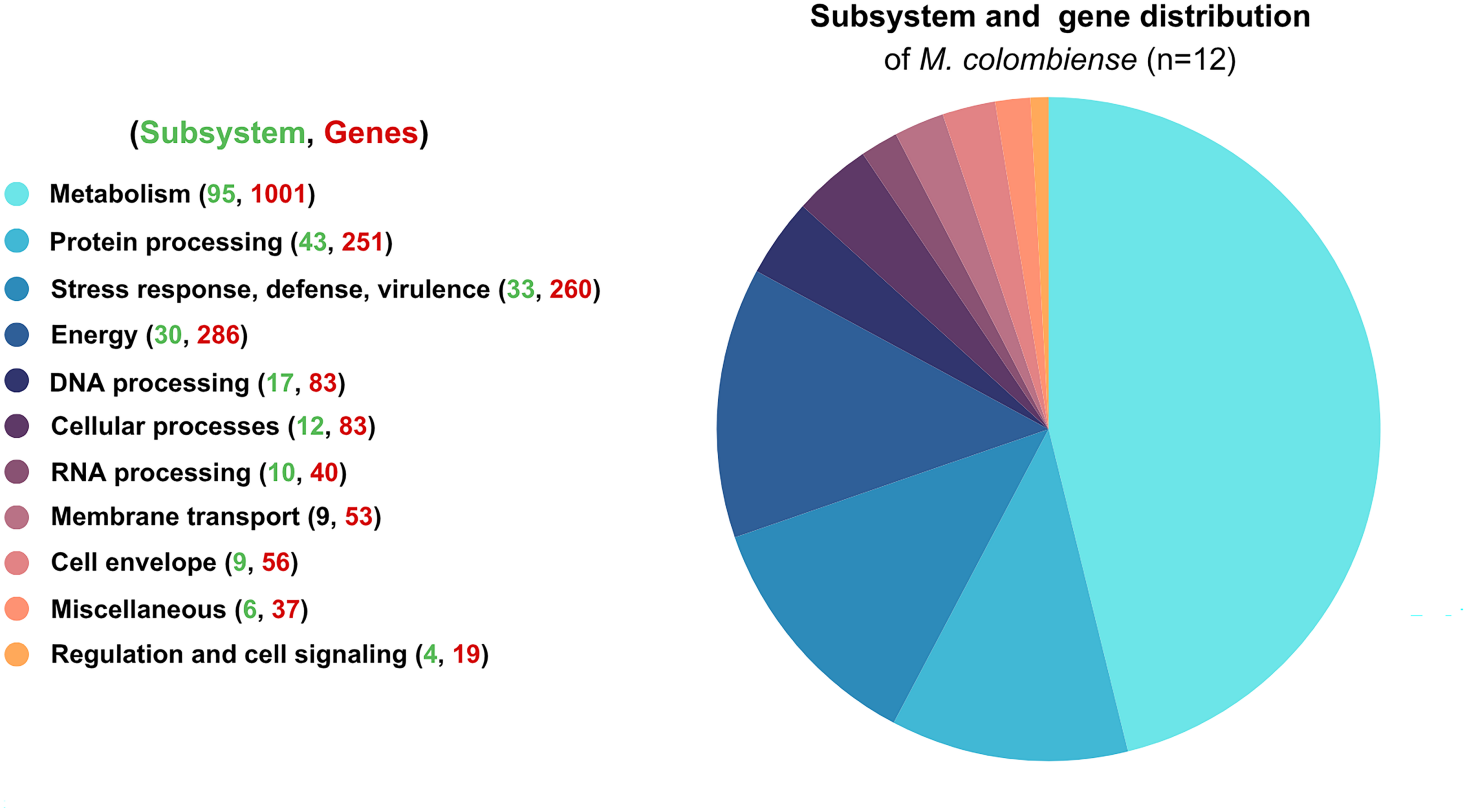
Functional subsystem annotation of 12 *Mycobacterium colombiense* genomes. Subsystem and gene distribution based on the genome annotation of *M. colombiense* strains performed using the RAST toolkit *via* the Bacterial and Viral Bioinformatics Resource Center platform.

Functional categorization revealed that most genes were involved in metabolism, followed by energy production, stress response, defense mechanisms, virulence, and protein processing. These distributions highlight the organism’s adaptability and survival strategies (Fig. 1).

### Antibiotic resistance, virulence factors, and CRISPR-Cas profiling

**Figure 2:**
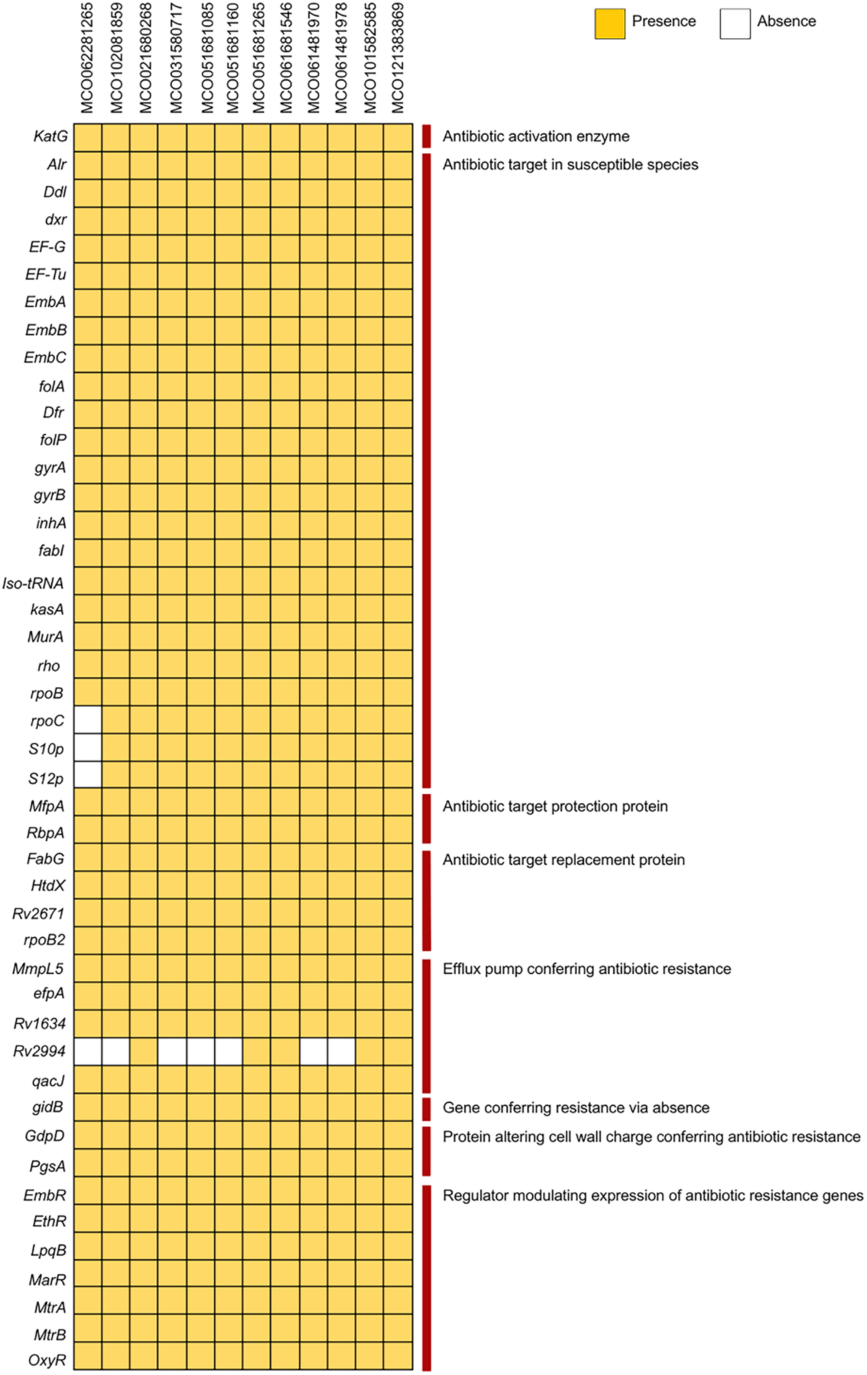
Distribution of antibiotic resistance genes among 12 *Mycobacterium colombiense* clinical isolates. Gene presence determined using the Bacterial and Viral Bioinformatics Resource Center, the Comprehensive Antibiotic Resistance Database *via* ABRicate, and the Resistance Gene Identifier. Yellow indicates gene presence; white indicates gene absence.

Analysis using the PATRIC and BV-BRC pipelines with the CARD database *via* ABRicate revealed the presence of multiple resistance-associated genes, including those related to antibiotic target modification (*alr, ddl, dxr, EF-G, EF-Tu, embA, embB, embC*), activation (*katG*), protection (*mfpA*), and target replacement (*fabG, htdX*). Efflux pump genes (*mmpL5, Rv1634, mtrA, efpA*), cell wall modification genes (*gdpD, pgsA*), and various regulatory genes (*embR, ethR, lpqB, marR, mtrA, mtrB, oxyR*) were broadly distributed. The *Rv2994* gene was absent in some isolates (Fig. 2, Table S3). Targeted interrogation of canonical resistance loci using RGI/CARD detected no resistance-associated mutations.

**Figure 3:**
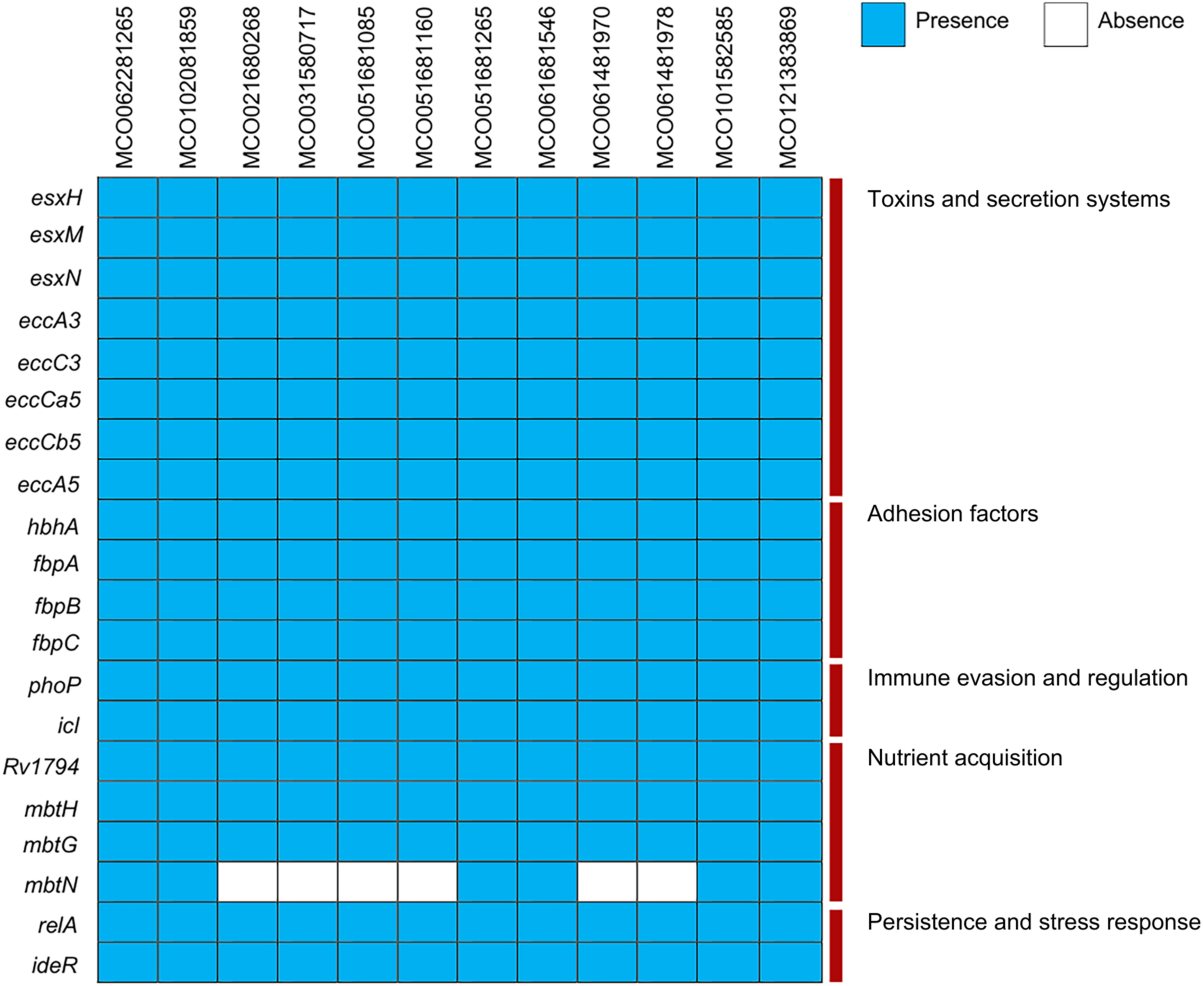
Distribution of virulence factors across 12 *Mycobacterium colombiense* clinical isolates. Gene presence determined using the Virulence Factor Database *via* ABRicate. Blue indicates gene presence; white indicates gene absence.

Virulence gene analysis using VFDB showed conserved presence of genes associated with toxin production, ESX secretion systems (*esxH, esxM, esxN, ecc*), adhesion (*hbhA, fbp*), immune evasion (*phoP, icl*), and nutrient acquisition (*mbtN*, absent in two isolates). Stress- and persistence-related genes (*relA, ideR*) were universally present (Fig. 3, Table S3).

CRISPR-Cas system analysis revealed variability in the number of CRISPR loci among isolates, ranging from six to nine per genome.

### Mutations in genes associated with phenotypic drug resistance

For amikacin, resistance is typically associated with mutations in *rrs*. No *rrs* mutations were detected in any *M. colombiense* isolates. For clarithromycin, mutations in *rrl* confer macrolide resistance. In this study, *rrl* mutations at positions A2270G/C (A2059G/C based on *E. coli* numbering) were identified exclusively in clarithromycin-resistant isolates.

For linezolid, resistance-associated mutations were assessed in *rplC* and *rplD*. Because all included isolates were linezolid-resistant, comparisons between susceptible and resistant groups could not be performed. Six synonymous mutations were identified in *rplC*, and eight mutations were detected in *rplD*, including one nonsynonymous substitution (Val218Ala).

For moxifloxacin, mutations within the quinolone resistance–determining regions (QRDRs) of *gyrA* and *gyrB* were analyzed. Several mutations were identified; however, the absence of moxifloxacin-susceptible isolates prevented definitive correlation between genotype and phenotype. Notably, three GyrA substitutions (Ala218Val, Val453Ile, Gly833Ser) and two GyrB substitutions (His250Arg, Thr522Ala) were observed (Tables 4, S4 and S5).

**Table 4:** Nucleic acid mutations and amino acid substitutions associated with amikacin, clarithromycin, linezolid, and moxifloxacin resistance in *Mycobacterium colombiense* clinical isolates.

Drug	Gene	Nucleic acid mutations found in resistant isolate (position on gene of a reference)^a^
Amikacin	*rrs*	Not found
Clarithromycin	*rrl*	A2270G/C **(A2059G/C)**^b^
Linezolid	*rplC*	C216G, T240C, T241C, C264T, T273C, C591G
	*rplD*	C96T, C207T, C309T, C411G, T447G, G489C, T621C, T653C
Moxifloxacin	*gyrA*	C54G, C144T, C183T, G216C, G234C, A357G, C381G, T477C, G486A, C543G, A639G, C653T, C693A, C696T, A792G, C795T, G804C, A822G, G861T, C1023T, C1056G, C1107T, G1143C, T1230C, G1242C, A1248G, T1273C, C1341G, G1357A, G1398A, C1407T, G1503C, T1512C, C1524T, C1602G, A1653G, C1698T, C1704T, C1740G, C1806A, A1905G, G1911A, T1993C, C2020T, C2025G/T, T2049G, C2064T, G2115A, C2154T, C2157T, C2205G, C2349G, C2385T, C2388G, G2397C, C2403T, T2424C, C2437T, C2493T, G2497A, G2511C
	*gyrB*	C216T, T240C, G270C, C318T, C357T, G387C, C399G, C402G, T489C, C543T, C555T, C576T, T586C, G615A, T651C, G693T, A741G, T744C, A749G, T756C, C759T, C852T, C894T, G909C, G981C, G984C, C1020G, G1101A, A1176G, G1239C, C1254G, C1266G, C1269A, G1311A, C1332G, C1341T, G1356A, C1386T, T1389C, C1398T, T1401C, C1413T, C1437G, C1512T, A1533G, C1542T, C1545T, C1551T, A1554G, A1564G, T1575C, G1629A, T1737C, C1749T, T1761C, T1812C, C1827T, T1893C, C1965G, G1968A
**Drug**	**Protein**	**Amino acid substitutions resistant isolate** **(position on gene of a reference)**^**a**^
Linezolid	50S ribosomal protein L3	Not found
	50S ribosomal protein L4	V218A
Moxifloxacin	GyrA	A218V, V453I, G833S
	GyrB	H250R, T522A

**Notes:**

a Position on gene of a reference based on *Mycobacterium colombiense* CECT 3050 (NZ_AFVW02000005.1).

b *Escherichia coli* numbering: A2059G/C mutations in bold are concordant with published studies.

### SNP-based phylogenetic tree

**Figure 4:**
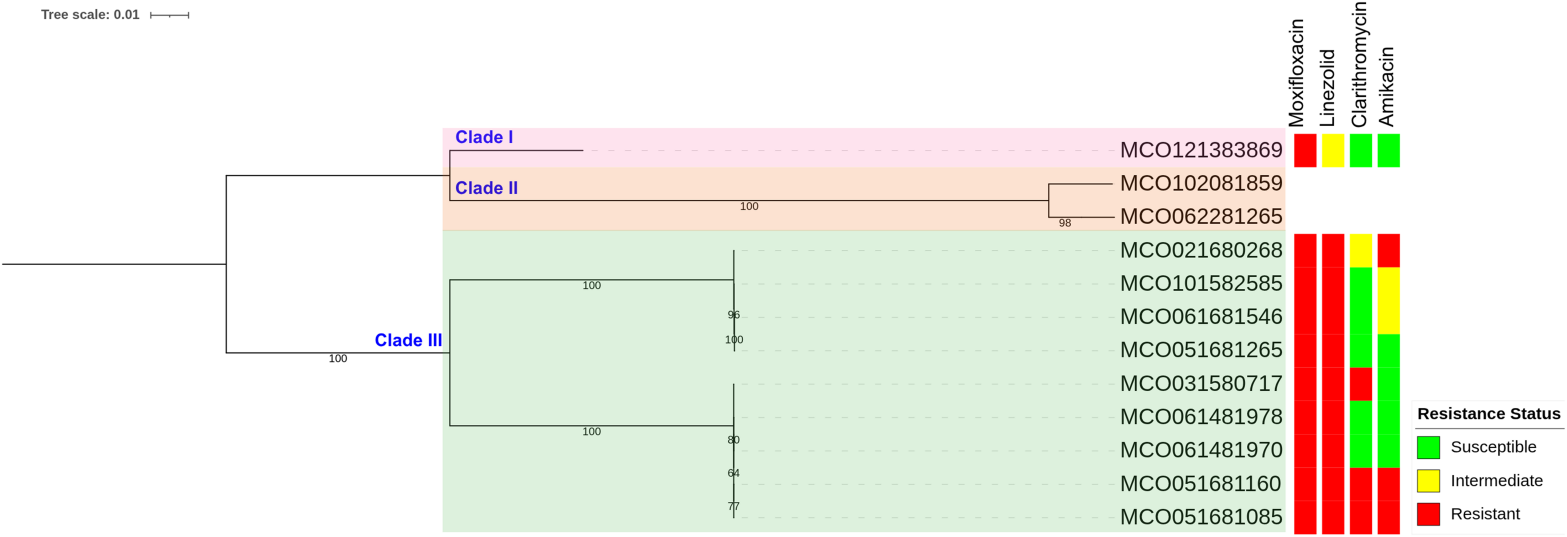
Maximum-likelihood phylogenetic tree and susceptibility profiles of four drugs in 12 *Mycobacterium colombiense* clinical isolates. Phylogenetic tree constructed using single nucleotide polymorphism-based data and visualized with iTOL software; a bootstrap consensus tree was inferred from 1,000 replicates.

Phylogenetic reconstruction based on SNP data revealed three main clades among the 12 *M. colombiense* clinical isolates (Fig. 4). Clade divisions were made according to pairwise SNP distances (Table S6). Clade I comprised one isolate (MCO121383869) which exhibited median SNP distances of 43,009 and 52,145 from Clades II and III, respectively. Clade II comprised two isolates (MCO102081859 and MCO062281265) which had a median of 7,598 SNPs from each other and median distances of 43,009 and 62,434 SNPs from Clades I and III, respectively. Clade III comprised nine isolates with median SNP distances of 52,145 and 62,434 from Clades I and II, respectively. Within Clade III, two subgroups were evident: Subgroup 1 included MCO021680268, MCO101582585, MCO061681546, and MCO051681265, with median pairwise distances of 47 SNPs (range: 31–57). Subgroup 2 included MCO031580717, MCO061481978, MCO061481970, MCO051681160, and MCO051681085, with median pairwise distances of 8 SNPs (range: 3–16).

### Public genome retrieval and species confirmation

Species confirmation was performed on 30 *M. colombiense* genomes, comprising the 12 clinical isolates from the current study and the 18 publicly available genomes (Table S2). The genome analysis results from NTM-Profiler, Pathogenwatch, and GTDB-Tk were classified as *M. colombiense*. However, TYGS analysis using digital DNA–DNA hybridization (dDDH) values (species threshold ≥70%) indicated that only 16 genomes (12 from this study and 4 from public databases) exceeded 80% similarity (dDDH-d_4_), consistent with *M. colombiense* classification. FastANI analysis (species threshold ≥95%) revealed that the same 16 genomes had ANI values above 97% (Fig. S1). To ensure accurate representation, only genomes meeting both criteria (dDDH ≥70% and ANI ≥95%) were included in the pangenome analysis.

### Pangenome analysis

**Figure 5:**
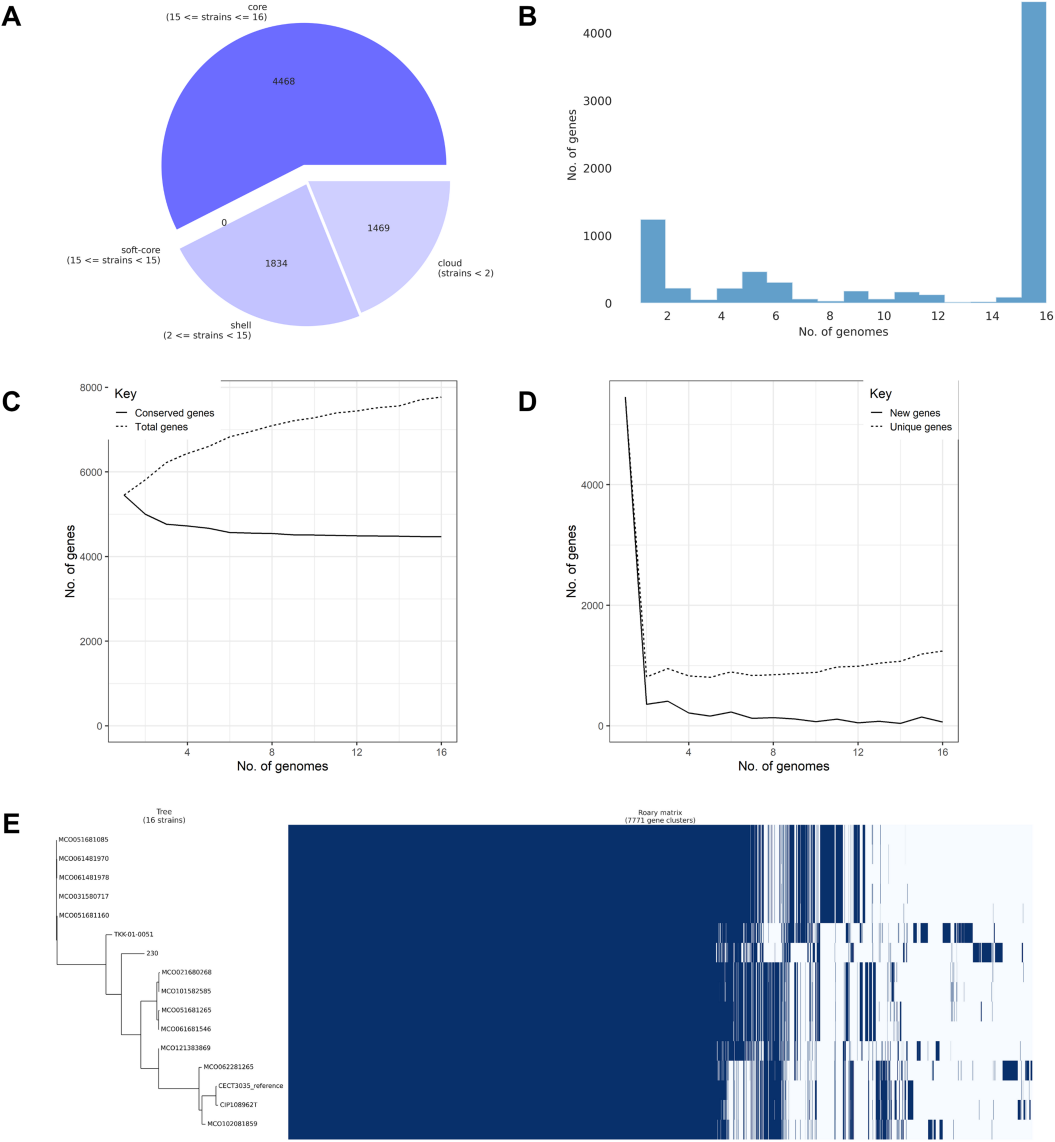
Pangenome analysis of 16 *Mycobacterium colombiense* genomes using Roary. (A) Composition of the pangenome, including 7,771 gene clusters categorized into core, accessory, and unique genes. (B) Gene frequency distribution across genomes: the x-axis represents the number of genomes sharing each gene; the y-axis indicates the number of genes at each frequency. Peaks at the left of the graph represent rare/unique genes; peaks at the right denote highly conserved genes. (C) Accumulation curve showing the total number of genes (dotted line) and conserved (core) genes (solid line) as genomes are sequentially added. (D) Reduction curve illustrating the number of new genes (solid line) and unique genes (dotted line) identified with each additional genome. In (C) and (D), the x-axis represents the number of analyzed genomes; the y-axis indicates the cumulative number of genes. (E) Presence–absence matrix of gene clusters across genomes, with blue indicating gene presence and white indicating absence. The matrix is aligned with a clustering dendrogram based on gene content similarity.

Pangenome analysis of the 16 confirmed *M. colombiense* genomes identified 7,771 gene clusters comprising 4,468 core genes (57.50%), 1,834 accessory genes (23.60%), and 1,469 unique or cloud genes (18.90%) (Fig. 5A). The high proportion of core genes confirmed a closed pangenome structure, indicating evolutionary conservation and low genetic variability among isolates. Gene cluster distribution (Fig. 5B) followed a typical pangenome pattern, with many genes shared across all genomes (core) and a smaller subset found only in a few genomes (accessory or strain-specific). The plateauing of the conserved gene curve (Fig. 5C) supports the closed pangenome structure, reflecting limited genetic acquisition. Conversely, the rapid decline in new gene discovery (Fig. 5D) indicates genomic stability across isolates, consistent with a conserved species. The gene presence–absence matrix and heatmap (Fig. 5E) suggests extensive gene content similarity among isolates, dominated by core genes. Only a small proportion of clusters varied between isolates, reflecting the low genomic diversity within the species.

**Figure 6:**
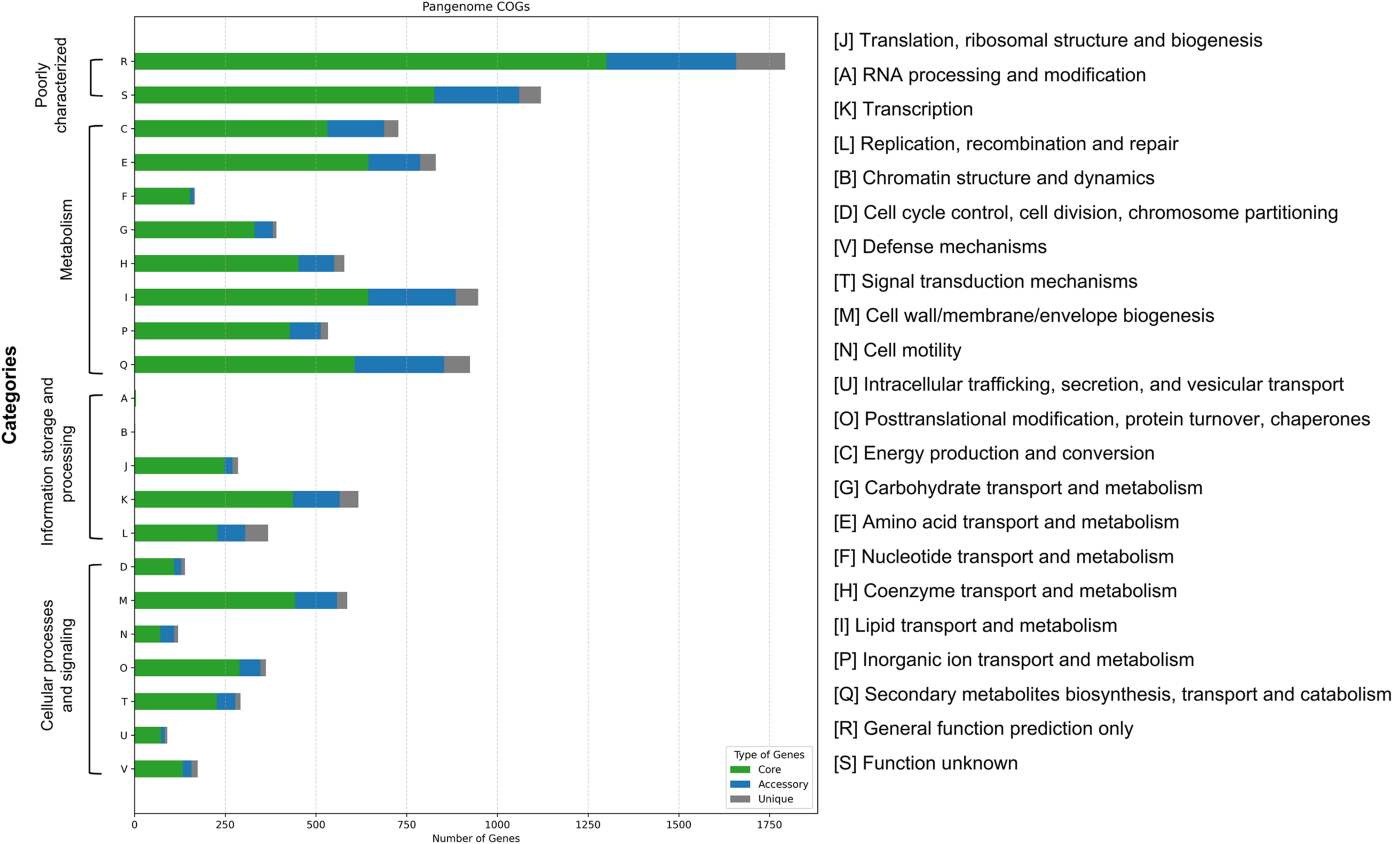
Functional categorization of pangenome genes in 16 *Mycobacterium colombiense* isolates based on Clusters of Orthologous Groups (COGs) analysis. Bar chart illustrates the distribution of core, accessory, and unique genes across 22 functional categories.

Functional classification using the COGs system revealed gene assignments across 22 categories (Fig. 6). Categories R (general function prediction only) and S (function unknown) were the most abundant across core, accessory, and unique gene groups, indicating a large proportion of uncharacterized functional genes. Among classified functions, Category E (amino acid transport and metabolism) was most prevalent in the core genome (645 genes), followed by Category I (lipid transport and metabolism, 643 genes) and Category Q (secondary metabolite biosynthesis, 605 genes). Accessory and unique genes were relatively enriched in Category Q, although their absolute numbers were significantly lower than those in the core genome. These findings suggest that while unique genes may contribute to niche-specific adaptations, most essential metabolic and functional pathways are conserved within the core genome.

## Discussion

*M. colombiense* has garnered increasing attention due to its association with chronic pulmonary infections, which are often challenging to diagnose and treat. Despite its clinical relevance, limited genomic data availability has hindered comprehensive understanding of its pathogenicity, antimicrobial resistance mechanisms, and epidemiological patterns. Recent advances in next-generation sequencing and bioinformatics tools have enabled more in-depth genomic investigations. In particular, comparative genomics and pangenome analysis facilitate the identification of core and accessory genes influencing phenotypic traits such as virulence, antimicrobial resistance, and environmental adaptability. Given the paucity of prior research on *M. colombiense*, the genomic insights presented in this study provide a valuable foundation for future investigations and may inform the development of targeted diagnostic tools and therapeutic strategies.

Although *M. colombiense* is a recently described species (first identified in 2006), available susceptibility data suggest behaviors similar to other MAC members (Pena et al., 2019). Prior studies report general susceptibility to macrolides, rifamycins, and aminoglycosides (Barretto et al., 2016; Benson et al., 2003; Maurer et al., 2019; Pena et al., 2019). In this study, drug susceptibility testing revealed high resistance rates to moxifloxacin and linezolid based on CLSI guidelines. These findings are consistent with previous studies reporting elevated resistance among *Mycobacterium* spp. to fluoroquinolones and oxazolidinones (Brown-Elliott, Nash & Wallace, 2012; Griffith et al., 2007), underscoring the importance of isolate-specific susceptibility testing to guide effective treatment. Moderate resistance to clarithromycin and amikacin (30% each) suggests their potential clinical utility, depending on isolate-specific susceptibility profiles. While a macrolide (clarithromycin or azithromycin) plus ethambutol and a rifamycin remains the recommended treatment regimen for MAC infections, individualized therapy guided by susceptibility testing is essential to optimize outcomes and prevent further resistance (Barretto et al., 2016; Qin & Tang, 2024).

The average genome size of the 12 *M. colombiense* isolates (5.79 Mbp) was slightly larger than the reference strain CECT 3035 (5.60 Mbp) (Gonzalez-Perez et al., 2016). All isolates possessed three rRNA genes, consistent with typical *M. colombiense* genomes; however, some NTM species have been found to possess more than two copies of rRNA genes (Takeda et al., 2018). This, together with the slight variation in tRNA gene counts, indicates underlying genomic plasticity. CRISPR loci ranged from six to nine, reflecting strain-specific differences in phage defense mechanisms and mobile genetic element exposure (Faure et al., 2019).

Despite increasing knowledge, the association between genotypic findings and phenotypic drug susceptibility remains incompletely resolved. No canonical resistance-associated mutations were detected in this study using curated homology and SNP models. The findings only partially explain the observed phenotypic resistance. While the genomic analysis suggests that these isolates may harbor resistance-associated genes, the limited coverage of existing databases constrains interpretation of the results. Importantly, gene presence alone does not confer resistance; specific mutations often determine functional resistance. This phenomenon is exemplified in *M. tuberculosis*, where *katG* mutations confer isoniazid resistance and *embB* mutations confer ethambutol resistance (Fenner et al., 2012).

The high prevalence of linezolid resistance (approximately 90%) may reflect alterations in ribosomal proteins (*e.g*., *rrl*, *rplC*, *rplD*) or regulatory genes, as reported in previous studies (Gan, Ng & Ngeow, 2023; Kim et al., 2019; Negatu et al., 2023). In our study, multiple mutations were identified among linezolid-resistant isolates. Six synonymous mutations were detected in *rplC*, and eight mutations were found in *rplD*, including one nonsynonymous substitution, Val218Ala (V218A). Although these mutations may contribute to linezolid resistance, their functional significance remains unclear and requires further investigation. In addition, the widespread presence of ribosomal protection and regulatory genes (*e.g*., *marR*, *mtrA*, *embR*) among the isolates supports the potential for complex resistance mechanisms. Non-ribosomal mechanisms, such as modifications in the mycolic acid synthesis pathway (*fadD32*) or efflux pump overexpression, have also been implicated (Gan, Ng & Ngeow, 2023).

The universal resistance to moxifloxacin is consistent with the presence of genes involved in DNA gyrase and topoisomerase function (*gyrA*, *gyrB*) as well as efflux pump activity (Kim et al., 2018; Yamaba et al., 2019). Several mutations were identified in *gyrA* and *gyrB*, including three amino acid substitutions in GyrA—Ala218Val (A218V), Val453Ile (V453I), and Gly833Ser (G833S)—and two substitutions in GyrB—His250Arg (H250R) and Thr522Ala (T522A). However, the specific roles of these mutations in moxifloxacin resistance in *M. colombiense* remain unknown. Uncharacterized target alterations or efflux overexpression may contribute to this resistance and warrant further investigation.

Moderate resistance to amikacin and clarithromycin (approximately 30%) may involve efflux pumps (*mmpL5*, *Rv1634*, *mtrA*, *efpA*), protective factors (*mfpA*), and target-modifying enzymes (*EF-G*, *EF-Tu*, *embA/B/C*). High-level aminoglycoside resistance typically involves mutations in *rrs*—such as A1408G, C1409T, and G1419T—commonly observed in kanamycin-resistant *M. tuberculosis* (Suzuki et al., 1998). Although no such mutations were identified among our isolates, alternative mechanisms may contribute to aminoglycoside resistance.

For clarithromycin resistance, mutational analysis of drug-target genes revealed that *rrl* mutations at A2270G/C were detected exclusively in clarithromycin-resistant isolates. These correspond to A2059G/C in *E. coli* numbering, which are well-established clarithromycin resistance–associated mutations in MAC and other NTM species (Bermudez et al., 2000; Huh et al., 2019; Meier et al., 1994; Nash & Inderlied, 1995). Thus, *rrl* mutations at A2059G/C are likely responsible for clarithromycin resistance in *M. colombiense*. These findings suggest that amikacin and clarithromycin remain generally effective against *M. colombiense*; however, the resistance observed in a subset of isolates underscores the importance of continuous surveillance and species-specific drug susceptibility testing to ensure effective therapeutic strategies.

Virulence factor profiling revealed a diverse array of genes implicated in intracellular survival, adhesion, immune modulation, and environmental persistence. Notably, conserved components of the ESX secretion system (*e.g*., *esxH*, *esxM*, *esxN*, *ecc* genes), adhesion factors (*hbhA*, *fbpA/B/C*), and regulatory elements (*phoP*, *icl*, *relA*, *ideR*) were consistently present, highlighting mechanisms shared with other MAC species and *M. tuberculosis* (Forrellad et al., 2013). These findings align with prior research on *M. colombiense* which identified secretion system-related genes as the largest functional group among virulence factors (Gonzalez-Perez et al., 2016). Moreover, this study corroborates prior observations regarding the partial loss of the ESX-1 locus: genes specifically associated with ESX-1 (*e.g*., *esxA*, *esxB*, *eccB1*, *eccCa1*, *eccD1*) were absent, whereas ESX-3 and ESX-5 components (*esxH*, *esxM*, *esxN*, *eccA3*, *eccC3*, *eccCa5*, *eccCb5*, *eccA5*) were universally present across isolates. While the presence of these genes suggests considerable pathogenic potential, their precise functional roles remain to be elucidated. Similar virulence and resistance gene patterns are reported in *M. avium* (Wanjiru et al., 2025). Comparative analysis with *M. tuberculosis* and *M. avium* revealed conserved virulence genes (*fbpA*, *fbpB*, *icl*, *ideR*, *relA*, *esxH*, *phoP*) alongside unique *ecc* genes, indicating shared pathogenic mechanisms and potential strategies for immune evasion. Partial loss of the ESX-1 secretion system is documented in other slowly growing mycobacteria, including *M. ulcerans* and *M. intracellulare* (Newton-Foot et al., 2016). The widespread presence of resistance-associated genes involved in target modification (*alr*, *ddl*, *EF-G*, *EF-Tu*, *embA/B/C*), activation (*katG*), protection (*mfpA*), and efflux mechanisms (*mmpL5*, *efpA*, *Rv1634*, *Rv2994*) indicates possible contributions to resistance mechanisms. This pattern is consistent with observations in other MAC species and *M. tuberculosis* (de Oliveira & Balan, 2020; da Silva et al., 2011; Gygli et al., 2017).

SNP-based phylogenetic analysis revealed three major clades, suggesting close genetic relatedness between the isolates. Although all isolates originated from human clinical samples, the presence of distinct clades may reflect geographic, environmental, or host-specific evolutionary pressures. While SNP-based phylogenetic *M. colombiense* studies remain limited, similar trends have been observed in other MAC species, in which genomic divergence is influenced by environmental adaptation (Mizzi et al., 2022).

Pangenome analysis of 16 confirmed *M. colombiense* genomes identified 7,771 gene clusters comprising 4,468 core genes, 1,834 accessory genes, and 1,469 unique (cloud) genes. The predominance of core genes supports the notion that this species maintains a relatively stable genome, consistent with observations in other bacterial species. The limited number of accessory and unique genes may reflect species-specific adaptations rather than frequent gene acquisition (Tettelin & Medini, 2020). This finding is consistent with studies on certain NTM species, such as *M. kansasii* (Sur, 2023); however, many NTM exhibit open pangenomes (Fedrizzi et al., 2017). Given the small sample size in this study, future investigations including a larger number of isolates are necessary to draw more robust and generalizable conclusions.

Functional classification using the COGs system assigned genes across 22 categories. Genes involved in lipid transport and metabolism (Category I) were predominant in both core and accessory genomes, reflecting the importance of lipid pathways in maintaining the mycobacterial cell envelope and mediating drug resistance (Jacobo-Delgado et al., 2023). A substantial proportion of genes were in Categories R (general function prediction only) and S (function unknown), particularly among unique genes, highlighting the potential for novel function discovery (Galagan et al., 2013; Tatusov et al., 2000). The enrichment of secondary metabolite biosynthesis genes (Category Q) among unique genes may contribute to environmental adaptation and antimicrobial compound production (Ghaly et al., 2023).

Despite these valuable insights, this study has limitations. First, the relatively small number of isolates and limited geographic representation may reduce the generalizability of the findings; studies with larger, more diverse sample sets are required to draw stronger and more representative conclusions. Second, the reliance on short-read sequencing restricts genome resolution; future studies incorporating long-read sequencing in future studies could help resolve structural variations and close repetitive genomic regions. Third, the interpretation of resistance-associated mutations is constrained by the absence of phenotypic drug susceptibility data for the reference genome (*M. colombiense* CECT 3035) and by the lack of susceptible isolates for certain antibiotics (*e.g*., linezolid and moxifloxacin), which limits the ability to distinguish true resistance determinants. Finally, the lack of functional validation of identified genes in this study, limited inferences regarding their roles in pathogenesis or environmental survival. Nonetheless, this is the largest investigated *M. colombiense* genome collection.

## Conclusions

This study presents a comprehensive pangenome analysis of *M. colombiense*, demonstrating substantial genetic diversity and a closed pangenome structure. The predominance of core genes underscores the species’ evolutionary conservation and low genetic variability among isolates. Drug susceptibility testing revealed high resistance to moxifloxacin and linezolid, with moderate resistance to clarithromycin and amikacin. The consistent presence of antibiotic resistance and virulence-associated genes highlights *M. colombiense* as a resilient and opportunistic pathogen capable of surviving in hostile environments and evading host immune responses, particularly in immunocompromised individuals. These findings provide valuable insights into the genomic diversity, antimicrobial resistance, and virulence potential of *M. colombiense* isolates to guide clinical management and inform the development of targeted diagnostic tools and infection treatment strategies.

## Supplemental Information

10.7717/peerj.20716/supp-1 Supplemental Information 1 Heatmap of average nucleotide identity (ANI) values among 30 *Mycobacterium colombiense* genomes. ANI percentages are illustrated using a color gradient from white (lower similarity) to red (higher similarity).

10.7717/peerj.20716/supp-2 Supplemental Information 2 Supplementary Tables.

## Additional Information and Declarations

### Data Availability

The following information was supplied regarding data availability:

The raw sequences are available at NCBI GenBank: PRJNA1271787.
