# Supplementary figures and images for: Whole-genome and pangenome insights into Mycobacterium colombiense clinical isolates from human infections
Source: PeerJ. 2026 Feb 2;14:e20716. doi: 10.7717/peerj.20716 (PMC12875251; doi:10.7717/peerj.20716)

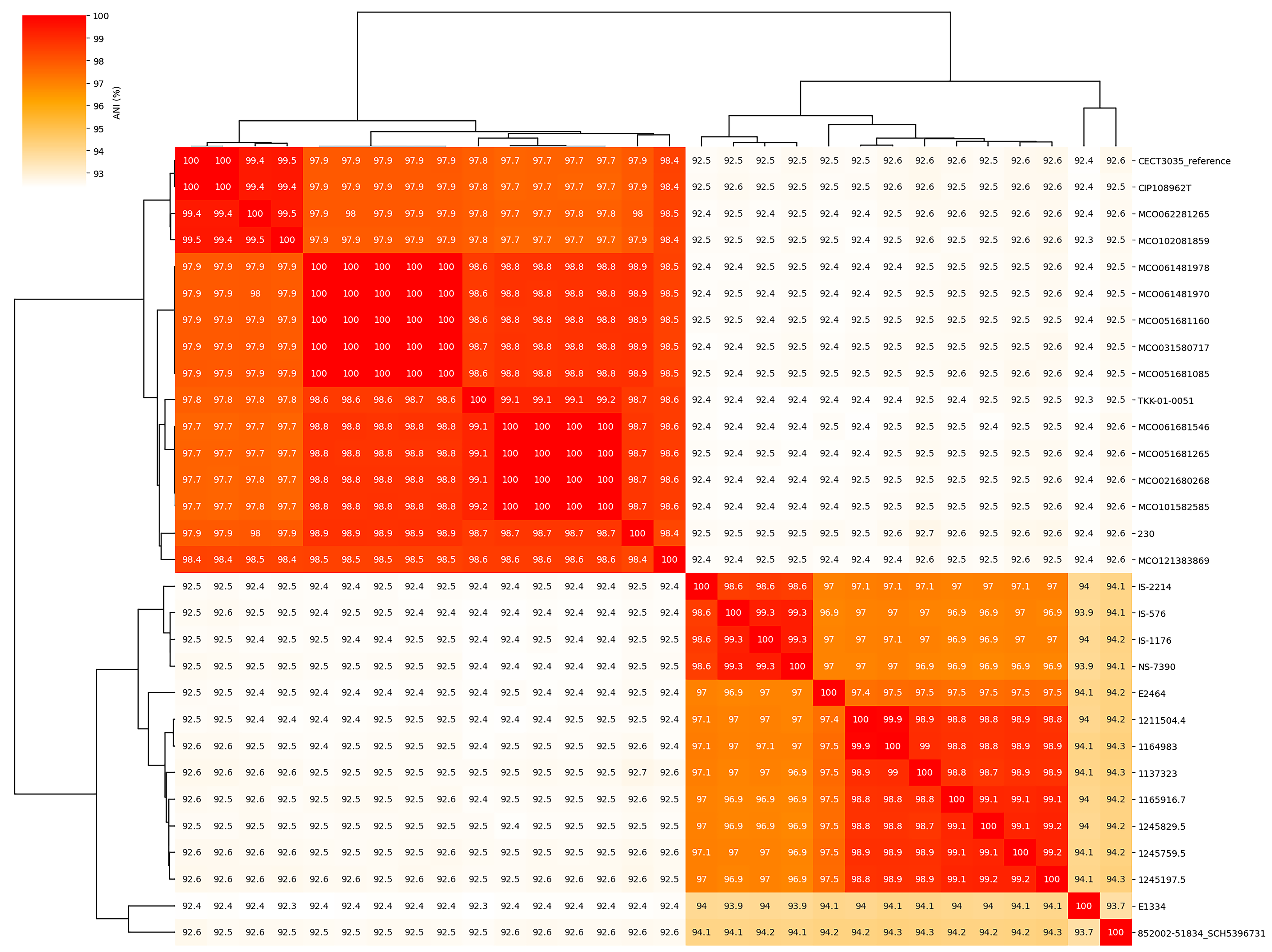

Supplement: Supplemental Information 1 — ANI percentages are illustrated using a color gradient from white (lower similarity) to red (higher similarity). [file peerj-14-20716-s001.png]
